# Pulses of labile carbon cause transient decoupling of fermentation and respiration in permeable sediments

**DOI:** 10.1002/lno.12411

**Published:** 2023-07-31

**Authors:** Philipp A. Nauer, Adam J. Kessler, Puspitaningsih Hall, Maria Elena Popa, Sophie ten Hietbrink, Tess Hutchinson, Wei Wen Wong, Karl Attard, Ronnie N. Glud, Chris Greening, Perran L. M. Cook

**Affiliations:** ^1^ Water Studies School of Chemistry, Monash University Clayton Victoria Australia; ^2^ School of Earth Atmosphere and Environment, Monash University Clayton Victoria Australia; ^3^ Institute for Marine and Atmospheric research Utrecht (IMAU), Utrecht University Utrecht Netherlands; ^4^ Nordcee and HADAL, Department of Biology University of Southern Denmark Odense M Denmark; ^5^ Danish Institute for Advanced Study, University of Southern Denmark Odense M Denmark; ^6^ Department of Ocean and Environmental Sciences Tokyo University of Marine Science and Technology Tokyo Japan; ^7^ Department of Microbiology Biomedicine Discovery Institute Clayton Victoria Australia

## Abstract

Dihydrogen (H_2_) is an important intermediate in anaerobic microbial processes, and concentrations are tightly controlled by thermodynamic limits of consumption and production. However, recent studies reported unusual H_2_ accumulation in permeable marine sediments under anoxic conditions, suggesting decoupling of fermentation and sulfate reduction, the dominant respiratory process in anoxic permeable marine sediments. Yet, the extent, prevalence and potential triggers for such H_2_ accumulation and decoupling remain unknown. We surveyed H_2_ concentrations in situ at different settings of permeable sand and found that H_2_ accumulation was only observed during a coral spawning event on the Great Barrier Reef. A flume experiment with organic matter addition to the water column showed a rapid accumulation of hydrogen within the sediment. Laboratory experiments were used to explore the effect of oxygen exposure, physical disturbance and organic matter inputs on H_2_ accumulation. Oxygen exposure had little effect on H_2_ accumulation in permeable sediments suggesting both fermenters and sulfate reducers survive and rapidly resume activity after exposure to oxygen. Mild physical disturbance mimicking sediment resuspension had little effect on H_2_ accumulation; however, vigorous shaking led to a transient accumulation of H_2_ and release of dissolved organic carbon suggesting mechanical disturbance and cell destruction led to organic matter release and transient decoupling of fermenters and sulfate reducers. In summary, the highly dynamic nature of permeable sediments and its microbial community allows for rapid but transient decoupling of fermentation and respiration after a C pulse, leading to high H_2_ levels in the sediment.

Sandy permeable sediments (permeability > 10^−12^ m^2^) occur globally along continental shelves and have been recognized as important drivers of ocean biogeochemical cycles (Huettel et al. 2014). The rapid turnover of organic matter in these sediments is largely driven by the deep (cm to dm scale) penetration of oxygen and other nutrients into the surface layers, due to an advection‐dominated transport regime caused by waves and tidal flows interacting with ripples and other topographical features. A dynamic transition zone exists between the fully oxic surface and the permanently anoxic subsurface, where frequent oxic–anoxic shifts occur on a regular tidal timescale (Cook et al. 2007; Glud 2008; McGinnis et al. 2014; Ahmerkamp et al. 2017). Due to their proximity to land, permeable sediments are often exposed to high nutrient and carbon (C) loadings near urban or agricultural centers. The high biological activity, nutrient, and C turnover in permeable sediments provide numerous ecosystem services. Yet, the pathways of C and nutrient degradation in permeable sediments are not well understood.

In cohesive sediments, organic matter degradation follows the redox cascade, which reflect a relatively stable and spatially separated succession of metabolic processes in order of their Gibbs free energy (Canfield et al. 2005). In such settings, hydrogen (H_2_) has been identified as a central intermediate and energy carrier between processes and subgroups of the microbial community (Hoehler et al. 1998). The high energy content and high diffusivity of H_2_ leads to tight spatiotemporal coupling of production and consumption processes. As a consequence, in situ concentrations of H_2_ converge toward the thermodynamic limit of the predominant terminal electron acceptor used for H_2_ oxidation (Hoehler et al. 1998) and generally range in the order of 1–6 nM. Recent studies have found hydrogen accumulation in permeable marine sediments well above thermodynamic expectations (Kleiner et al. 2015; Bourke et al. 2016; van Erk et al. 2020), which has been ascribed to the decoupling of fermentation and sulfate reduction (Kessler et al. 2019).

Thus far, the underlying causes for the decoupling of fermentation and respiration in permeable sediments has remained elusive. Two main hypothesis were put forward: (i) frequent oxic–anoxic shifts cause generalist bacteria such as facultative fermenters to dominate over specialist sulfate reducers, thus leading to H_2_ accumulation under anoxic conditions (Kessler et al. 2019; Chen et al. 2022); (ii) frequent physical disturbance, that is, particle resuspension and migration events, leads to removal of microbial consortia disrupting established closely coupled interactions between fermenters and sulfate reducers, which could lead to H_2_ accumulation (Kessler et al. 2019). To discriminate these possibilities, we tested if oxic–anoxic shifts, the physical disturbance of sediment, or pulses of labile C cause decoupling and H_2_ accumulation. Our overall objectives were (i) to survey the extent and prevalence of elevated H_2_ concentrations in near‐shore waters and permeable sediments and (ii) to investigate potential prerequisites for H_2_ accumulation under controlled laboratory conditions.

## Materials and methods

### Field sites

Permeable sediment and water‐column sampling was conducted near shore in the subtidal zone (~ 1 m depth at low tide) at four field sites in Australia (Middle Park Beach, 37.855°S/144.962°E; Inverloch Pier, 38.636°S/145.734°E; Werribee Southern Beach, 37.970°S/144.703°E; Heron Island, 23.442°S/151.910°E), and two field sites in Denmark (Hjerting Badehotel, 55.522°N/8.353°E; Fællestrand, 55.610°N/10.612° E). These sites were chosen because they were the focus of previous studies in fermentation in permeable sediments (Middle Park Beach and Heron Island), have high nutrient loading (Werribee Southern Beach), have extensive sandbanks exposed and inundated over a tidal cycle (Inverloch Pier), provide a carbonate sediment contrast (Heron Island) to silicate sediment (all other sites), and provide a temperate and geographic contrast (Hjerting Badehotel and Fællestrand) to the Australian sites. All sampling and processing at Hjerting Badehotel and Fællestrand were conducted in September 2019, and at Heron Island in November 2018, June 2019, and January 2020. At Middle Park Beach, cores for sediment gas profiles were collected and processed in December 2019, while water‐column sampling and in situ processing were carried out during a 5‐d period in February 2020. At this site, we also collected permeable sediment samples for column flow‐through reactors in November 2019 and a flume experiment in September 2020. At Werribee Southern Beach, cores for sediment gas profiles were collected and processed in July 2020. Samples for slurry incubations were collected at various sites and dates (Supporting Information Table S1).

### Water‐column time series

Sampling locations for water‐column time series were at or near the mouth of an inlet or reef, thus integrating over a large area of permeable sediment. Samples were processed in the field to avoid chemical water preservation and storage, which can potentially result in H_2_ contamination (Novelli et al. 1987). Water was collected at 0.5–1 m water depth using a horizontal hand‐operated Niskin bottle from a jetty at Middle Park Beach, Inverloch Pier, and Heron Island, or manually from beyond the wash zone at Hjerting Badehotel and Fællestrand. Triplicate 160‐mL serum vials were filled from the bottom up with a gas‐tight tube, with approximately 300 mL left overflowing; the vial was then closed with a black butyl rubber stopper pinched with a needle, which was removed immediately to avoid any initial gas phase in the vials. Using two gas‐tight plastic syringes, a 20‐mL ultrapure N_2_ headspace was introduced by simultaneously removing 20 mL liquid. Serum vials were then shaken vigorously for 2 min and left to equilibrate for 5 min; 17 mL of headspace was then extracted into a N_2_‐flushed syringe with stopcock by reinjecting the extracted liquid. After purging the stopcock and needle with 2 mL sample, 15 mL sample gas was injected into a N_2_‐flushed and evacuated silicone‐closed Exetainer, then sealed with stainless steel bolt and O‐ring (Nauer et al. 2021). The employed method for gas extraction and sample processing was validated by processing seawater purged with a calibration gas of known H_2_ concentration. Blanks of N_2_ gas were collected and stored in the same way. Tests showed negligible differences in H_2_ contamination between procedural blanks of N_2_‐purged seawater and field‐processed N_2_ gas, indicating that no H_2_ contamination originates from transferring the headspace sample into evacuated silicone‐closed Exetainers.

At selected sites and time points, we sampled additional seawater from the Niskin flask for H_2_ stable isotope analysis (Kessler et al. 2019). In brief, 500 mL seawater was drawn into a 1.6‐liter polycarbonate syringe and equilibrated with a 1.1‐liter headspace of ambient air. The headspace was then transferred into clean pre‐evacuated 1‐liter borosilicate glass flasks with two valves (Normag). The flasks have polychlorotrifluoroethylene sealing and are stable for H_2_ over several years (Jordan and Steinberg 2011).

After gas‐sample processing, water temperature, salinity, and O_2_ concentration were measured in the remaining water in the Niskin using a Hach probe (HQ40d), or the data were obtained from a remote lander containing a multiparameter probe (Seabird SBE 19 Plus V2 with an Aanderaa 4340 O_2_ optode) deployed nearby at Hjerting Badehotel and Fællestrand.

Mixing ratios of H_2_ were measured on a gas chromatograph with a pulsed discharge helium ionization detector (model TGA‐6791‐W‐4U‐2, Valco Instruments Company Inc.; Islam et al. 2019). Final concentrations *C*_*i*,*w*_ of dissolved gas *i* (H_2_ or CH_4_) in seawater were calculated via mass balance:

(1) $$C_{i,w}=\frac{C_{i,g}\left(V_{g}+H_{i}V_{w}\right)}{V_{w}}$$

Here, *C*_*i*,*g*_ is the molar gas concentration in the headspace, converted from the measured mixing ratio via ideal gas law; *V*_*g*_ and *V*_*w*_ are the respective headspace and water volume; and *H*_*i*_ denotes the dimensionless air–water partitioning coefficient for the respective gas, adjusted for salinity and temperature (Sander 2015).

The samples for H_2_ stable isotopic composition (δD) were analyzed by a continuous flow isotope ratio mass spectrometry method (Rhee et al. 2004; Chen et al. 2015) and calibrated against a gas standard of known H_2_ mole fraction (550 ppb) and δD (+ 95‰ Vienna Standard Mean Oceanic Water). The isotope measurements have a typical uncertainty better than 2‰.

### Sediment depth profiles

Similar to water‐column samples, in situ porewater profiles of dissolved H_2_ and O_2_ were obtained from sediment cores processed in the field. Sediment cores for collecting porewater consisted of acrylic tubes of 67 mm inner diameter and 5 mm wall thickness, featuring 5 mm diameter holes (ports) drilled into the side at 2 cm spacing and sealed with soft silicone. After collecting the core, the top bung was removed and a stainless‐steel needle inserted sideways through the silicone ports. The custom‐made needle was sealed at the tip and had 10 evenly spaced 0.2‐mm side cuts to extract porewater through a sufficiently large area with minimal risk of clogging. The needle was connected to a 60‐mL gas‐tight plastic syringe and stopcock via a flow‐through optode to measure O_2_ (OXFTC2; Pyroscience GmbH). After discarding 5 mL, 40 mL porewater was carefully extracted with minimal suction to avoid vacuum, that is, allowing the water head to “push” porewater into the syringe without making a headspace. Subsequently, a headspace of 20 mL N_2_ was introduced directly into the syringe, equilibrated through shaking for 2 min, and the headspace was transferred and stored as described above for serum vials. Cores were processed from the top downwards, with the first sample consisting of the overlying water, followed by 3–4 sediment depths in 4–8 cm intervals. This avoided overlap between depths due to the large extracted volume. At Heron Island, only one sample per core could be collected at the deepest extraction port, as coarse coral debris limited the coring depth to approximately 10 cm.

### Slurry incubations

Three types of slurry experiments were performed to elucidate the key factors that could trigger potential microbial decoupling and H_2_ accumulation (Supporting Information Table S1). Experiment A investigated the effect of oxic periods in otherwise anoxic sediment; Experiment B compared undisturbed (still) and disturbed (shaken/suspended) sediment from different sites; and Experiments C1 and C2 investigated additions of organic carbon in conjunction with disturbance. Sediment was sampled from the subtidal zone using polycarbonate cores of 30 cm length. Depth horizons were generally mixed during preparation unless stated otherwise (Supporting Information Table S1). All slurries were set up in 160‐mL serum vials sealed with butyl rubber stoppers, using 30 g of sediment in 70 mL seawater for shaken and 70 g sediment in 30 mL seawater for still slurries. Assuming a porosity of 0.4 and sediment density of 1.92 g mL^−1^, the resulting headspace was approximately 74 and 94 mL, respectively. Oxic seawater was used for preparation, and slurries were turned anoxic by flushing the headspace with N_2_ for 5 min, shaking vigorously for 2 min, and flushing again for 5 min which was shown to make the slurries completely anoxic. However, some slurries in Experiment A were processed under fully anoxic conditions in the glovebox using deep (25–30 cm) sediment and anoxic seawater (Supporting Information Table S1). Slurries in Experiments C1 and C2 were prepared with artificial seawater (Kester et al. 1967), either with or without sulfate as the sole terminal electron acceptor present. Shaken slurries were placed horizontally on a rotary shaker at 130 rpm or as indicated otherwise (Supporting Information Table S1); still slurries were left upright. In treatments with added carbon, we used equal masses of glucose and spirulina to reach a concentration equivalent of 1 mM glucose in the vials; in selected vials, sodium molybdate was used at 20 mM to selectively inhibit sulfate reduction (Banat et al. 1983). Gas samples of 2 mL were extracted daily using gas‐tight plastic syringes with a stopcock after injecting 2 mL high‐purity N_2_ to replace extracted gas. The sample was transferred into N_2_‐flushed 3 mL silicone‐closed Exetainers and stored capped with bolt and O‐ring (Nauer et al. 2021). In Experiments C1 and C2, liquid samples were collected every second day by injecting 7 mL N_2_ beforehand, then extracting 3 mL liquid with a plastic syringe, transferred to a glass vial, and poisoned with 10 *μ*L 6% HgCl_2_ for dissolved inorganic carbon (DIC) analysis. Total DIC concentration was measured using an Apollo SciTech DIC analyzer. Furthermore, for dissolved organic carbon (DOC), approximately 20 mL of liquid sample was collected from the stock solutions at the start, and from the slurries at the end of the experiment, then filtered through 0.45‐*μ*m pore‐size filter and acidified for storage. DOC concentration was determined using a Shimadzu TOC‐5000 Total Organic Carbon analyzer.

### Flow‐through reactors

Anoxic flow‐through reactors were set up to investigate the effect of oxygen exposure and physical disturbance on H_2_ accumulation. Reactors comprised of short cylinders (4.4 cm inner diameter and 4.2 cm height) with channeled grooves in the end caps to allow even distribution of flow (Evrard et al. 2013). Five treatments with three replicates each were set up: (i) sampled undisturbed, left undisturbed; (ii) sampled disturbed, left undisturbed; (iii) sampled disturbed, regularly disturbed; (iv) sampled undisturbed, left undisturbed, inhibited sulfate reduction; (v) sampled disturbed, regularly disturbed, inhibited sulfate reduction. An empty control reactor was included. To allow physical disturbance of sand particles, flow‐through reactors were filled only half with 40 mL of sediment, resulting in approximately 2 cm of overlying water. These columns were manually shaken for 2 min once per day. For the six undisturbed flow‐through reactors, we sampled intact cores to 4 cm and scooped out the excess to reach 40 mL sediment volume. Other “sampled disturbed” flow‐through reactors were packed in the laboratory with 40 mL sieved and homogenized sediment. Flow was directed through the sediment from the bottom upwards, with a flow rate of 0.8 mL min^−1^ of recirculating seawater from the sampling site, using a peristaltic pump (Ismatec IPC‐16). Two separate reservoirs of 4 liters volume each, one without and one with 20 mM sodium molybdate to inhibit sulfate reduction, were constantly purged with a steady flow of high‐purity N_2_ to keep them anoxic. The reservoirs were recirculating but replaced with fresh anoxic seawater once a day. Oxygen at the outlet of the reservoirs and outlet of two selected reactors was monitored with a flow‐through optode (OXFTC2; Pyroscience GmbH). After 10 d of anoxia, a brief oxic phase was introduced for 24 h to investigate short term exposure to oxygen; the total duration of the experiment was 17 d.

Sampling for dissolved H_2_ was conducted once per day, approximately 3 h after changing the reservoir to allow flow‐through reactor volumes to be replaced twice. Gas‐tight 60‐mL plastic syringes with a stopcock were connected to the flow‐through reactor outlets, and approximately 45 mL porewater was collected. After discarding excess sample to reach 40 mL, a headspace of 20 mL N_2_ was introduced into the syringe, which was shaken for 30 min on a shaker table, then left to equilibrate for 30 min; 15 mL of the headspace was then transferred into a 12 mL pre‐evacuated silicone‐closed Exetainer and sealed for subsequent gas analysis. Sampling and processing took approximately 2 h; a test with calibration gas showed negligible H_2_ turnover in the syringes during processing. Gas concentrations were measured as described above. Production rates of H_2_ were calculated by multiplying the flow rate by the concentration change observed in the reactor (inlet–outlet).

### Flume experiment

To investigate H_2_ accumulation after a C pulse under near‐field conditions, we conducted a flume experiment with permeable sediment from Middle Park Beach site A. The custom‐built flume tank has been described earlier (Kessler et al. 2012). In brief, ~ 11 liters of sediment were filled into a central lower part of the rectangular tank, and ~79 liters of water were circulated above with an electric boat propeller at a constant flow rate of 5.4 cm s^−1^. Ripples of ~2–3 cm height were sculpted by hand at the start of the experiment to induce typical flow patterns across the sediment–water interface (Kessler et al. 2012). The experiment ran for 14 d. After an initial equilibration phase of 6 d, a pulse of ~ 1 mM C in the form of food‐grade commercial spirulina powder was added to the water column on Day 7, and monitoring continued until the end of the experiment. The water column was constantly aerated, except during the daily ~ 2–2.5 h sampling procedure when gas exchange was reduced to diffusive atmospheric exchange. During these periods porewater and water‐column gas samples for H_2_ analysis were collected daily. Samples for DIC (both porewater and water column) and DOC (water column only) were collected every other day, as described above. Porewater was extracted from a grid of silicone‐sealed sampling ports at four positions (two troughs and two ripples) and three depths, each at 3 cm spacing, using the same procedure as for the in situ cores. Oxygen concentrations were determined using a planar optode on the reverse side of the sampling ports, once before and once after sampling (Kessler et al. 2012). The O_2_ exchange rate was determined once before the experiment by filling the tank with water of ~ 20% O_2_ saturation and monitoring O_2_ with a Hach DO probe during equilibration with ambient air. Furthermore, a blank test was conducted in the water‐filled flume to test for potential H_2_ contamination. After aerating for 24 h, aeration was stopped and triplicate water‐column samples were collected three times within 2 h for H_2_ extraction as described above.

## Results and discussion

### Hydrogen concentrations above permeable sediments

To investigate the extent and magnitude of potential H_2_ accumulation in waters above permeable sediment, time series of hourly or daily water‐column H_2_ concentrations were collected at five sites. At one site, seasonal dynamics were followed by sampling during two periods across an annual cycle. With the exception of the time series at Heron Island in November 2018, H_2_ concentrations remained below 16 nM, with a median of 1.9 nM and 1.6–3.6 nM interquartile range, and were in a similar range to previous observations in surface waters of Saanich Inlet, U.S.A. (Lilley et al. 1982) but generally higher than nearshore coastal waters (Kawagucci et al. 2014). This is above the atmospheric equilibrium in the water column, but near the expected thermodynamic equilibrium for hydrogeontrophic sulfate reduction in porewaters of marine sediment (Hoehler et al. 1998). Porewater profiles confirmed concentrations of H_2_ in the range of 0.7–18 nM, with a median of 4.6 nM (Supporting Information Fig. S1). At the targeted sites and time points, we found no evidence for H_2_ concentrations in the high nM to *μ*M range that would suggest microbial decoupling of fermentation and sulfate reduction and thus H_2_ accumulation (Fig. S1). It appears that, even in highly dynamic permeable sediments, H_2_ production and consumption remain closely coupled most of the time, and the high H_2_ concentrations in the upper nM to *μ*M range we reported previously (Bourke et al. 2016; Kessler et al. 2019; 2020) are exceptions rather than the norm. However, high H_2_ concentrations in the water column at Heron Island in November 2018 confirmed that H_2_ can occasionally reach levels orders of magnitude above the atmospheric or thermodynamic equilibrium for benthic sulfate reduction. The observation thus supports the hypothesis that H_2_ only accumulates during infrequent and transient decoupling events imposed by environmental triggers. H_2_ isotopic composition shows a depleted source; the source signature of −779‰ (calculated using a Keeling plot, Supporting Information Fig. S2) indicating high confidence of a H_2_O—H_2_ equilibrium (often interpreted as microbial origin), though it is not possible to differentiate between sources (Walter et al. 2012, 2013).

**Fig. 1:**
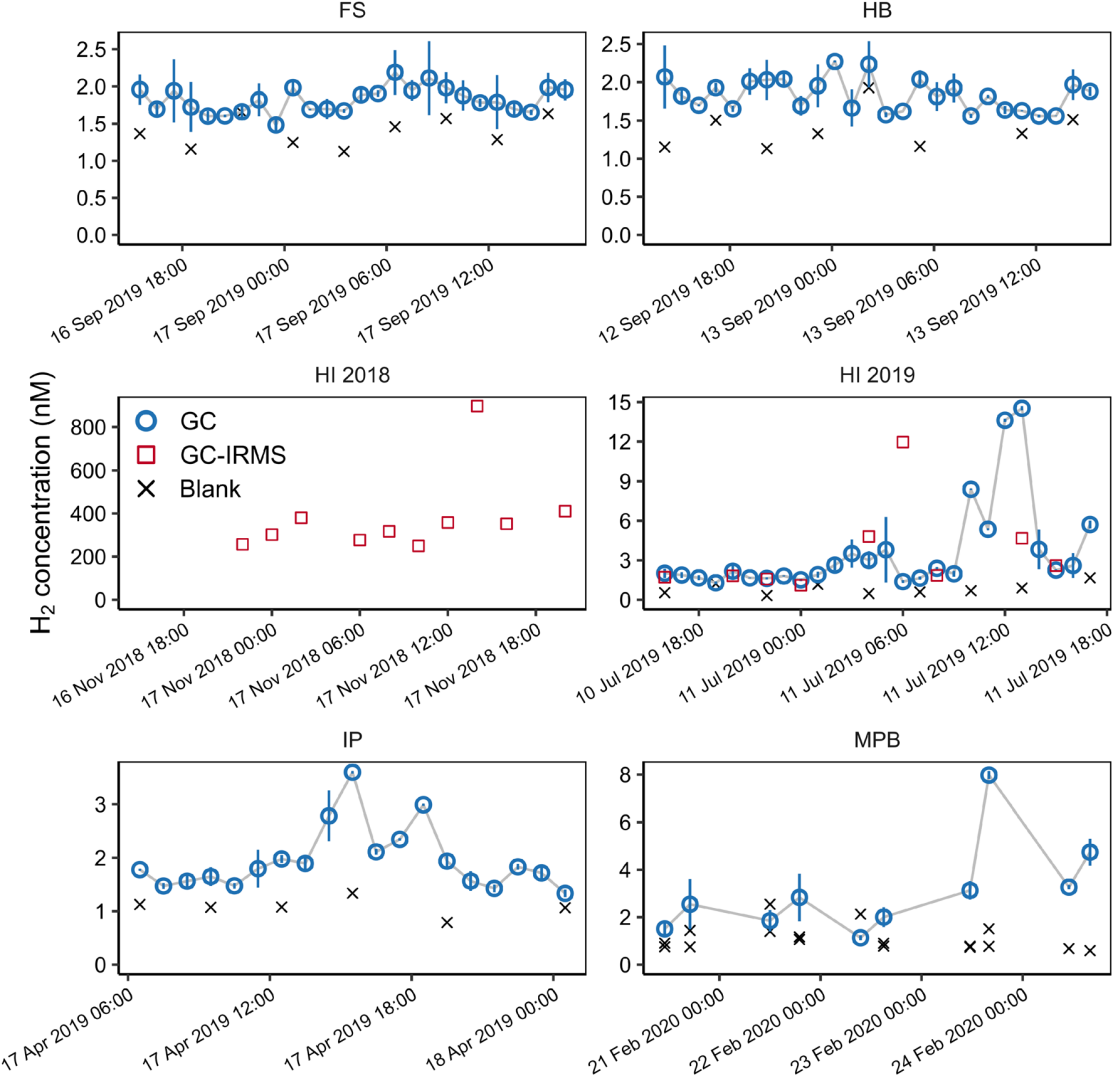
Time series of H_2_ concentrations in the water column at five different field sites: Hjerting Badehotel (HB), Fællestrand (FS), Heron Island (HI), Inverloch Pier (IP), Middle Park Beach (MPB). At MPB we sampled twice daily over 5 d; at other sites we sampled hourly over a full tidal cycle. Error bars depict standard error of the mean (*n* = 3). Note the different scale of the *y*‐axes.

Coincidentally, our water‐column sampling at Heron Island in November 2018 occurred during a coral spawning event, when concentrations of DOC around the reef can spike above background levels (Wild et al. 2004*a*,*c*; Glud et al. 2008). This likely triggered a rapid increase in aerobic C turnover (Wild et al. 2004*b*), followed by a massive increase in fermentation by facultative anaerobes that often dominate the microbial community in shallow permeable sediments (Kessler et al. 2019; Chen et al. 2022). This is consistent with the microbial H_2_ source indicated by isotopic composition. We thus argue that the observed oversaturation of H_2_ was a transient effect induced by a temporarily saturated sulfate reduction capacity, which is generally mediated by slow‐responding specialists and obligate anaerobes (Kessler et al. 2019; Chen et al. 2022). It is also possible that elevated hydrogen concentrations are derived from water‐column processes such as photochemistry (Punshon et al. 2007) or possibly within anoxic niches in agglomerations of suspended organic matter; however, the extent of oversaturation here strongly suggests sediment sources. In 2019 and 2020, we observed a lower degree of oversaturation at Heron Island (Fig. 1) and Keeling plots (Supporting Information Fig. S2) indicate different or mixed sources of H_2_. At other sampling sites and times, we found no indications of C inputs above background levels, for example, from a local algae bloom. Interestingly, we also did not detect H_2_ accumulation in porewater samples at Werribee Southern Beach (Supporting Information Fig. S1), although C and nutrient loading is generally high due to a large wastewater treatment plant in close proximity (Wong et al. 2022). We thus presume that only a sudden “spike” or pulse of C would trigger H_2_ accumulation, while constantly high C loads would lead to a new low‐concentration equilibrium, with the local sulfate reduction community adapted to such conditions. Our series of laboratory experiments (see below) support this mechanism of a pulsed C input as the most probable trigger for decoupling and H_2_ accumulation.

### Oxic–anoxic shifts do not affect hydrogen accumulation

**Fig. 2:**
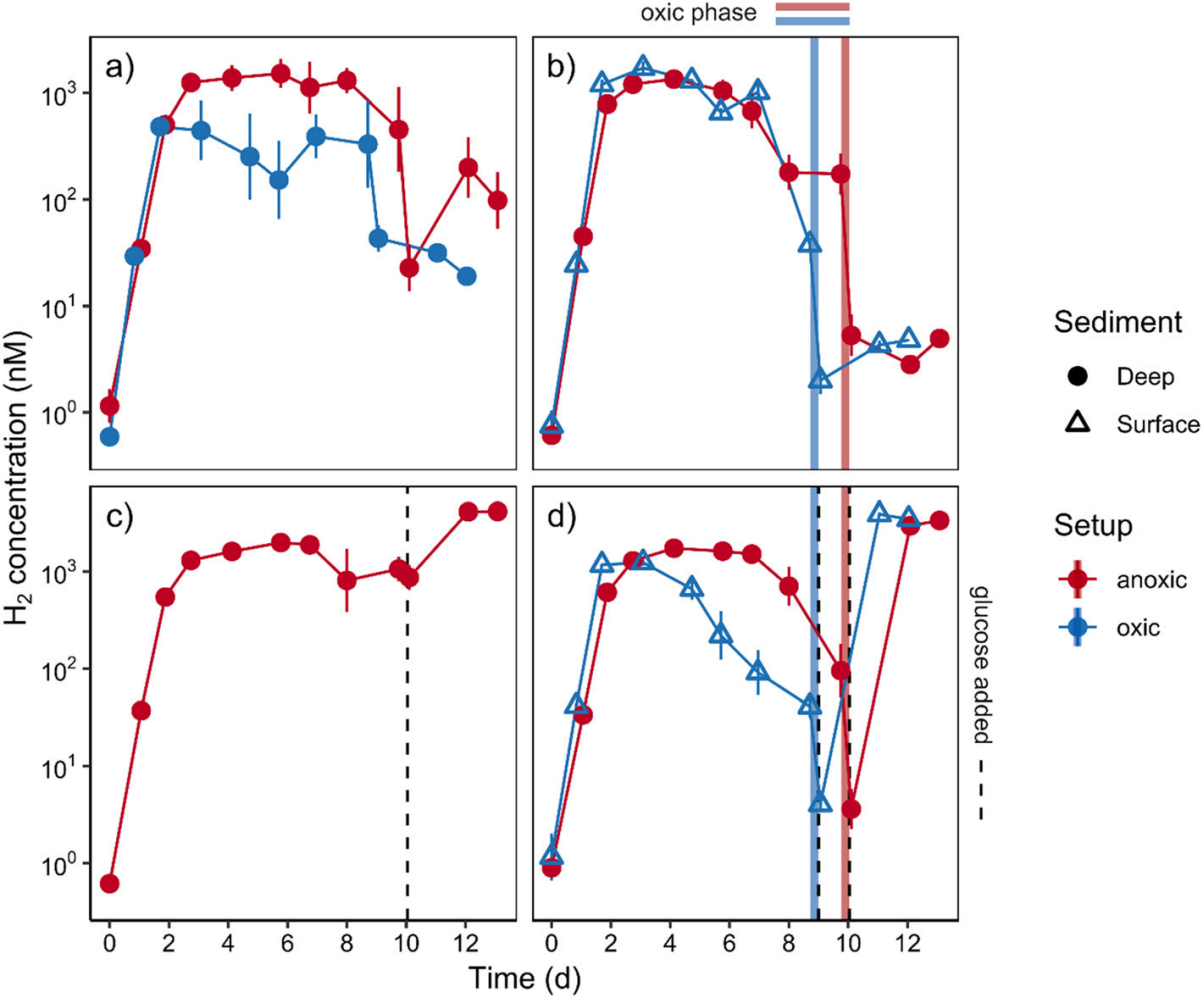
Hydrogen concentrations in slurry Experiment A with deep (> 20 cm) and surface (< 5 cm) permeable sediment (shapes) in the absence or presence of oxygen during setup (colors). (**a**) No oxic phase and no glucose addition during incubation; (**b**, **d**) oxic phase after 9–10 d (colored bars); (**c**, **d**) glucose addition after 9–10 d to alleviate potential C limitations after long incubations (dotted line). Error bars represent standard error of the log mean (*n* = 3). Note the logarithmic scale of the *y*‐axes.

We first tested the hypothesis of oxic–anoxic shifts acting as a potential trigger for decoupling respiration and fermentation in slurred sediment from deep (> 20 cm) anoxic sediment and surface (< 5 cm depth) sediment samples from Middle Park Beach (Experiment A, Supporting Information Table S1). Samples were either processed in the glovebox (anoxic setup) or in the lab in oxic seawater (oxic setup). Regardless of setup or sediment origin, all slurries showed strong and prolonged H_2_ accumulation over > 10 d (Fig. 2). In particular, deep and fully anoxic sediment without any exposure to oxygen in situ and during processing showed strong decoupling and H_2_ accumulation, similar to slurries with previous oxygen exposure. Furthermore, introducing an oxic phase of 6 h after 9–10 d of anoxia did not affect the ongoing rate of H_2_ accumulation (Fig. 2b). However, H_2_ concentrations rose rapidly in all slurries that received 1 mM of glucose, and this response was unrelated to O_2_ exposure (Fig. 2c,d). H_2_ accumulation after the addition of a C source has previously been observed in cohesive as well as permeable sediments (Kessler et al. 2019), an effect that most likely is attributed to stimulated fermentation.

**Fig. 3:**
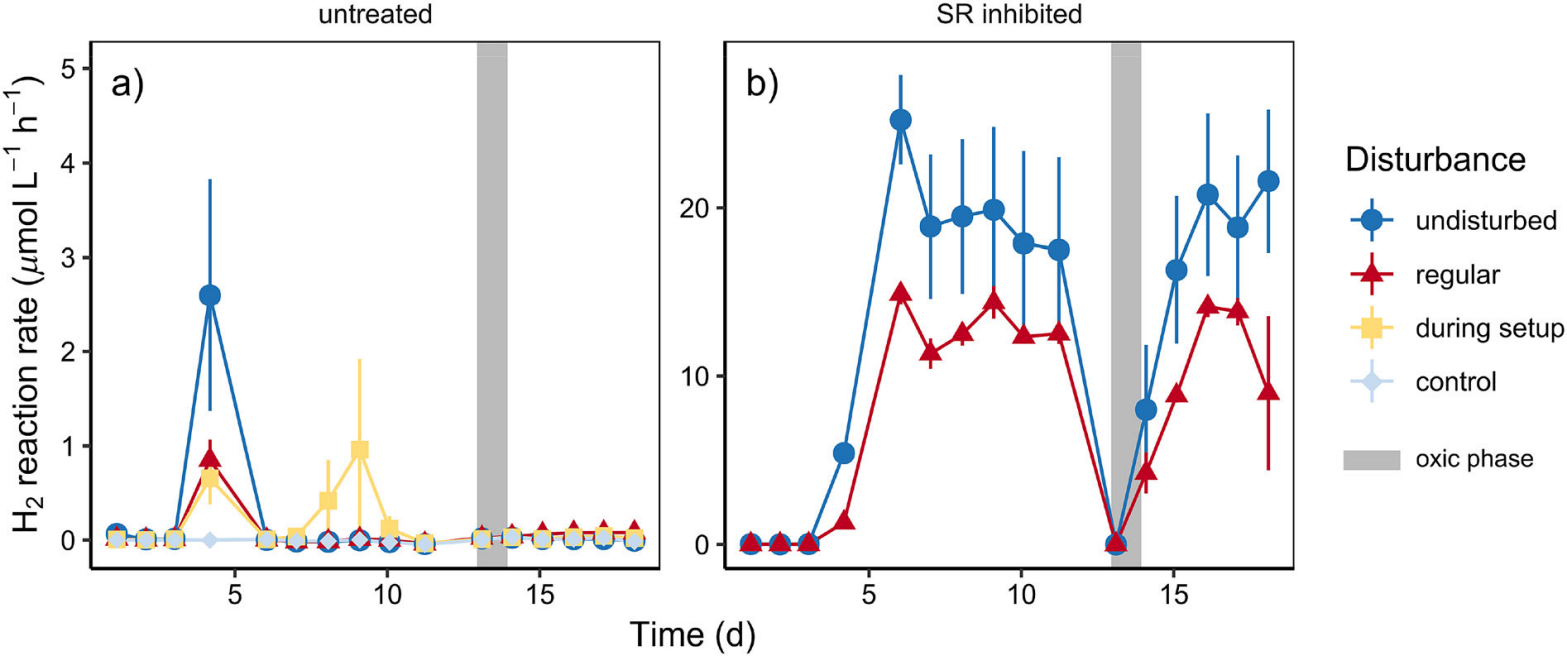
Rates of net H_2_ production in flow‐through reactors with an oxic phase after extended anoxia, (**a**) without and (**b**) with addition of molybdate to inhibit sulfate reduction (SR). Flow‐through reactors were set up in triplicates with different physical disturbance treatments. Error bars denote standard errors of the mean (*n* = 3). Note the different scales of the *y*‐axes. Undisturbed sediments were collected intact in the field, regular sediments were disturbed after column set up, during set up were disturbed by sieving and repacking sand into the columns, control is an empty flow‐through reactor.

To test if O_2_ exposure could trigger H_2_ accumulation under more realistic flow conditions, we set up flow‐through reactors and introduced an oxic phase after an initial anoxic stabilization period of about 2 weeks. In a subset of flow‐through reactors, sulfate reduction was inhibited by the addition of molybdate. Untreated flow‐through reactors showed small brief peaks of H_2_ production at the start of the experiment and low rates thereafter (Fig. 3a, consistent with previous results from similar flow‐through reactor experiments with Middle Park Beach sediment (Bourke et al. 2016; Kessler et al. 2019). After a short lag‐phase, molybdate‐inhibited flow‐through reactors showed high and persistent H_2_ production rates (Fig. 3b). This confirmed that, under extended anoxic conditions, fermentation remained active and sulfate reduction was the main sink for H_2_. In untreated flow through reactors the low H_2_ production rates remained unaffected by a 24 h period of O_2_ exposure (Fig. 3a), while they dropped and recovered rapidly in sulfate reduction‐inhibited flow‐through reactors with limited H_2_ sink capacity (Fig. 3b). Apparently, both fermenters and sulfate reducers in these sediments retained or rapidly recovered their activity after an extended oxic phase. Indeed, sulfate reducers in permeable sediments with transient O_2_ exposure have been shown to coexist with aerobes and facultative anaerobes (e.g., fermenters), and were able to retain their function (Saad et al. 2017). Hence, oxic–anoxic shifts or vice versa can apparently be ruled out as a trigger for microbial decoupling and H_2_ accumulation in the targeted settings.

### Physical disturbance and hydrogen accumulation

In addition to O_2_, we tested the effect of mild physical disturbance (brief daily resuspension) on H_2_ accumulation in the same flow‐through reactor experiment by comparing undisturbed flow‐through reactors packed in the field with flow‐through reactors disturbed once during setup or resuspended daily for 2 min. Contrary to our expectations, undisturbed and disturbed flow‐through reactors showed similar H_2_ production patterns (Fig. 3a). Regardless of inhibition of sulfate reduction by molybdate addition, undisturbed flow‐through reactors exhibited the highest rates and heterogeneity between replicates (Fig. 3a,b). Furthermore, resuspension during the oxic period did not lead to prolonged decoupling or stronger H_2_ accumulation. Decoupling of sulfate reduction and fermentation at early stages of the experiment was observed for all disturbance regimes and thus must have been caused by an unrelated trigger; in contrast, brief daily resuspension events appeared to have little effect on H_2_ dynamics.

**Fig. 4:**
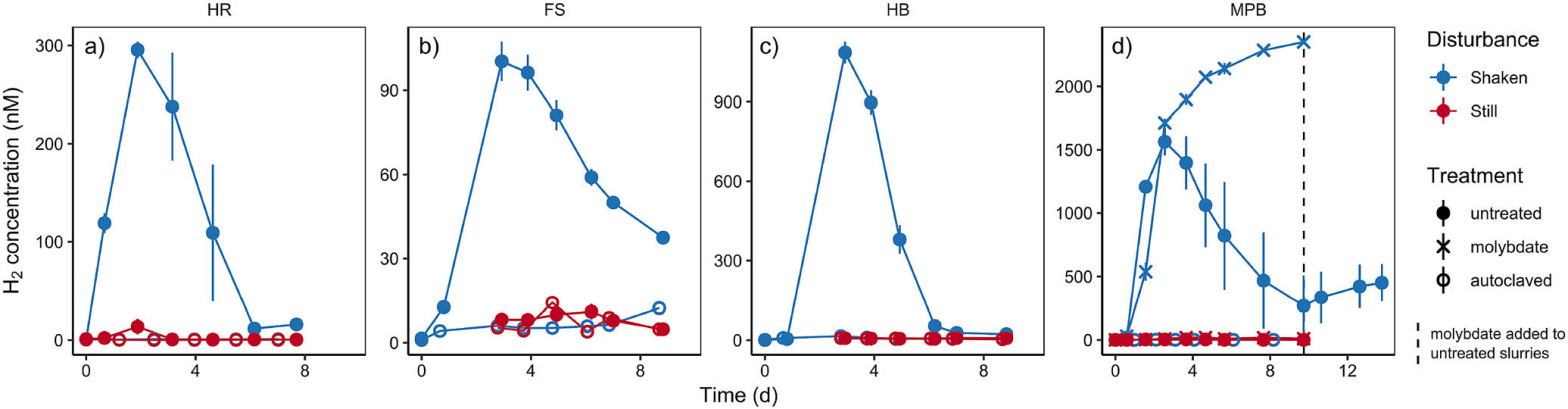
Hydrogen concentrations in slurry Experiment B with permeable sediment from various sites left still or shaken at 130 rpm. (**a**–**c**) Slurries from Heron Island (HR), Faellestrand (FS), and Hjerting Badehotel (HB); the headspace of these still slurries was sampled repeatedly without shaking. (**d**) Slurries from Middle Park Beach (MBP); these still slurries were set up in triplicates for each time point and sampled sacrificially after brief vigorous shaking, to exclude potential transport bias. For some Middle Park Beach treatments, sulfate reduction was inhibited from the start of the experiment or after 9 d by adding 20 mM molybdate. Error bars represent standard error of the mean (*n* = 3). Note the different *y*‐axis scales for different sites.

To better clarify the response to physical disturbance, we compared H_2_ concentrations between still and shaken sediment slurries of various origin (i.e., Heron Island, Fællestrand, Hjerting Badehotel, and Middle Park Beach; Fig. 4). All constantly shaken slurries produced excess H_2_ after 1–2 d of anoxia, a H_2_ concentration peak after 2–3 d, and net consumption after 3–4 d. This pattern was consistent across all four sites (although the amplitude varied by more than an order of magnitude) and is in agreement with previous results from shaken slurry experiments with Middle Park Beach sediments (Kessler et al. 2019). In stark contrast, none of the slurries left still showed H_2_ accumulation; fermentation and respiration remained closely coupled, with H_2_ concentrations fluctuating around a low level of 2–8 nM, which coincides with the thermodynamic equilibrium during coupled fermentation and sulfate reduction (Hoehler et al. 1998). It is unlikely that these low H_2_ headspace concentrations were caused by imperfect or delayed mixing with the still sediment, as still slurries with brief vigorous shaking just before sacrificial sampling (Fig. 4d) showed the same behavior as continuously still slurries (Fig. 4a–c). Still slurries thus appeared to behave more like cohesive sediments, while constant and vigorous shaking had a clear transient effect on H_2_ turnover, regardless of site. In essence, initially identical microbial communities can exhibit fundamentally different H_2_ turnover when exposed to physical disturbance. Yet, the contradictory results observed in our slurry and flow‐through reactor experiments hint toward another underlying factor that ultimately causes microbial decoupling between fermenters and sulfate reducers and thereby H_2_ accumulation.

To further elucidate disturbance effects, some slurries from MBP were treated with molybdate to investigate H_2_ turnover in the absence of sulfate reduction. Shaken slurries showed prolonged H_2_ accumulation when sulfate reduction was inhibited, with concentrations plateauing after approximately 6–8 d (Fig. 4d). Also, H_2_ concentrations in shaken slurries increased again when molybdate was added at a later stage, thus confirming the pattern observed in similar previous experiments that (delayed) sulfate reduction was largely responsible for the decrease in H_2_ (Kessler et al. 2019). In contrast, molybdate had no effect in still slurries; there was no detectable H_2_ accumulation even when sulfate reduction was inhibited (Fig. 4d). In turn, this suggests that H_2_ accumulation in shaken untreated slurries was not caused by a suppression of sulfate reduction; that is, physical disturbance does not seem to act on the H_2_ sink. Rather, the transient H_2_ accumulation consistently observed in shaken slurries was a consequence of stimulated fermentation and not suppression of respiration (i.e., sulfate reduction). This could stem from either an actual release of labile organic material caused by desorption of polymers due to particle collisions during shaking, or simply from better microbial access to labile organic material when transport limitations are lifted by shaking.

### Pulsed carbon inputs trigger hydrogen accumulation

**Fig. 5:**
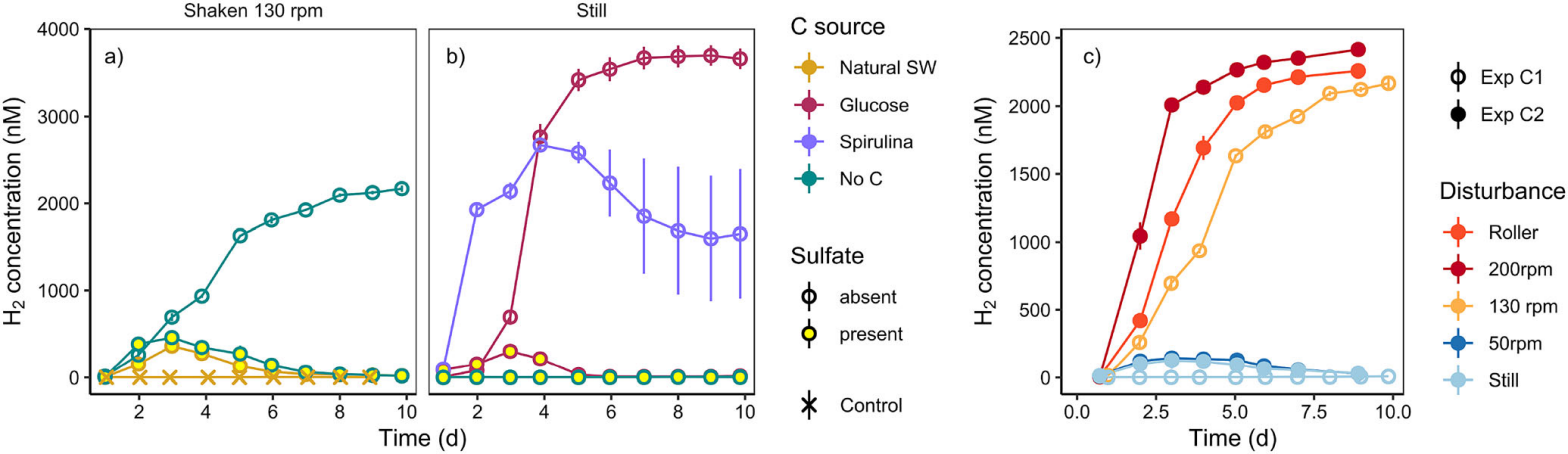
Hydrogen concentrations in still and shaken slurries of Experiments C1 (**a**, **b**) and C2 (**c**) with permeable sand from Middle Park Beach. Slurries were triple‐washed and prepared with artificial seawater (SW) with and without the presence of sulfate, leaving only particle‐bound organic matter as available organic carbon (C) source. Controlled additions of labile organic matter included preparation with natural SW (thus adding suspended and dissolved organic matter), and additions of glucose (1 mM) or spirulina powder (equal mass to glucose ~ 1 mM equivalent). For Experiment C2, all slurries were prepared with artificial SW without sulfate, and particle‐bound organic matter was the only source of organic material. The respective still and shaken treatments from Experiment C1 were added in this plot for comparison. Note that all slurries in Experiment C2, including the still treatment, were accidentally shaken at 200 rpm for 2 min during setup. Error bars represent standard error of the mean (*n* = 3).

In slurry Experiments C1 and C2, we tested if shaking of sandy sediment could lead to an actual release of labile organic material that then causes microbial decoupling of fermentation and sulfate reduction followed by H_2_ accumulation. This experiment aimed at complementing the observation where H_2_ accumulated after the addition of glucose (Experiment A), and the observation of stimulated fermentation in shaken slurries (Experiment B). Experiment C1 investigated H_2_ accumulation after addition of labile organic material in the form of glucose or spirulina in still and shaken slurries with artificial seawater. The set‐up enabled controlling the sources of organic material and terminal electron acceptors, primarily sulfate, available to microbes (Fig. 5). In agreement with Experiment B, shaken slurries showed H_2_ accumulation (Fig. 5a), while still slurries without addition of labile organic material did not (Fig. 5b), regardless of sulfate presence. When extra organic material was added, still slurries showed similar H_2_ accumulation patterns to shaken slurries without carbon addition (Fig. 5b). In the presence of sulfate, most of this H_2_ was consumed after Days 3–5, while it accumulated in the absence of sulfate. Hence, adding labile organic material to still slurries led to microbial decoupling and similar H_2_ accumulation patterns than those observed in all slurries shaken at 130 rpm. This suggests that regular and strong shaking leads to the release of labile organic material, causing rapid stimulation of fermentation and temporary saturation of sulfate reduction capacity. Near‐identical H_2_ accumulation in shaken slurries with artificial (organic‐carbon‐free) seawater and natural seawater indicates that particle‐bound organic material is mobilized during shaking. This interpretation was supported by a significant increase in DOC in most shaken slurries, while still slurries without addition or organic material retained initial DOC levels (Supporting Information Fig. S3). We presume that the frequent high‐energy collisions of sand particles can lead to mechanical disruption of biofilm and release of organic polymers that become susceptible to enzymatic hydrolysis. Interestingly, adding spirulina or glucose led to slightly different H_2_ accumulation patterns in the absence of sulfate (and other inorganic electron acceptors) (Fig. 5b), with a faster response of fermenters to spirulina. The nature of the carbon source thus appeared to influence H_2_ turnover, with a more natural mix of different C sources (such as in spirulina powder) promoting a more complex response.

Experiment C2 tested the effect of different levels of shaking on H_2_ accumulation in the absence of sulfate (Fig. 5c). Shaking frequency was positively correlated with the observed H_2_ production rates, with frequencies > 50 rpm showing rapid and sustained H_2_ accumulation in the first 2–3 d, until reaching a plateau after 5–8 d. A roller shaker had a similar effect to rotary shakers at high frequencies (> 50 rpm), that is, particles remained mostly suspended and in constant motion while shaking. At 50 rpm, particles remained settled at the bottom of the horizontally positioned serum vial while the overlying seawater was in constant motion, thus ensuring turbulent water mixing without particle suspension. Slurries shaken in this way (i.e., well‐mixed but without inducing particle collisions) showed only minimal H_2_ accumulation, identical to still samples (Fig. 5c). DOC measurements at the end of the experiment confirmed that no additional organic matter was released from still slurries and slurries shaken < 50 rpm, while DOC concentrations in other shaken treatments were 3–4 times higher than initial values (Fig. S3). Therefore, particle‐bound C was mobilized by particle collisions when shaking sandy slurries above a threshold frequency (> 50 rpm) that led to regular (re‐)suspension of most particles. In summary, the sudden availability of labile organic matter is the common denominator for all observed instances of decoupling and H_2_ accumulation, with the exception of flow‐through reactor experiments. In this particular experiment, we speculate that a potential mass die‐off of strictly aerobic organisms may have released labile organic matter after a few days of induced anoxia, although this remains to be confirmed experimentally. It is also possible that phages could contribute to cell lysis releasing organic matter (Carreira et al. 2013). Hence, available evidence points toward a pulse of labile organic matter as the main cause for decoupling in permeable sediments.

**Fig. 6:**
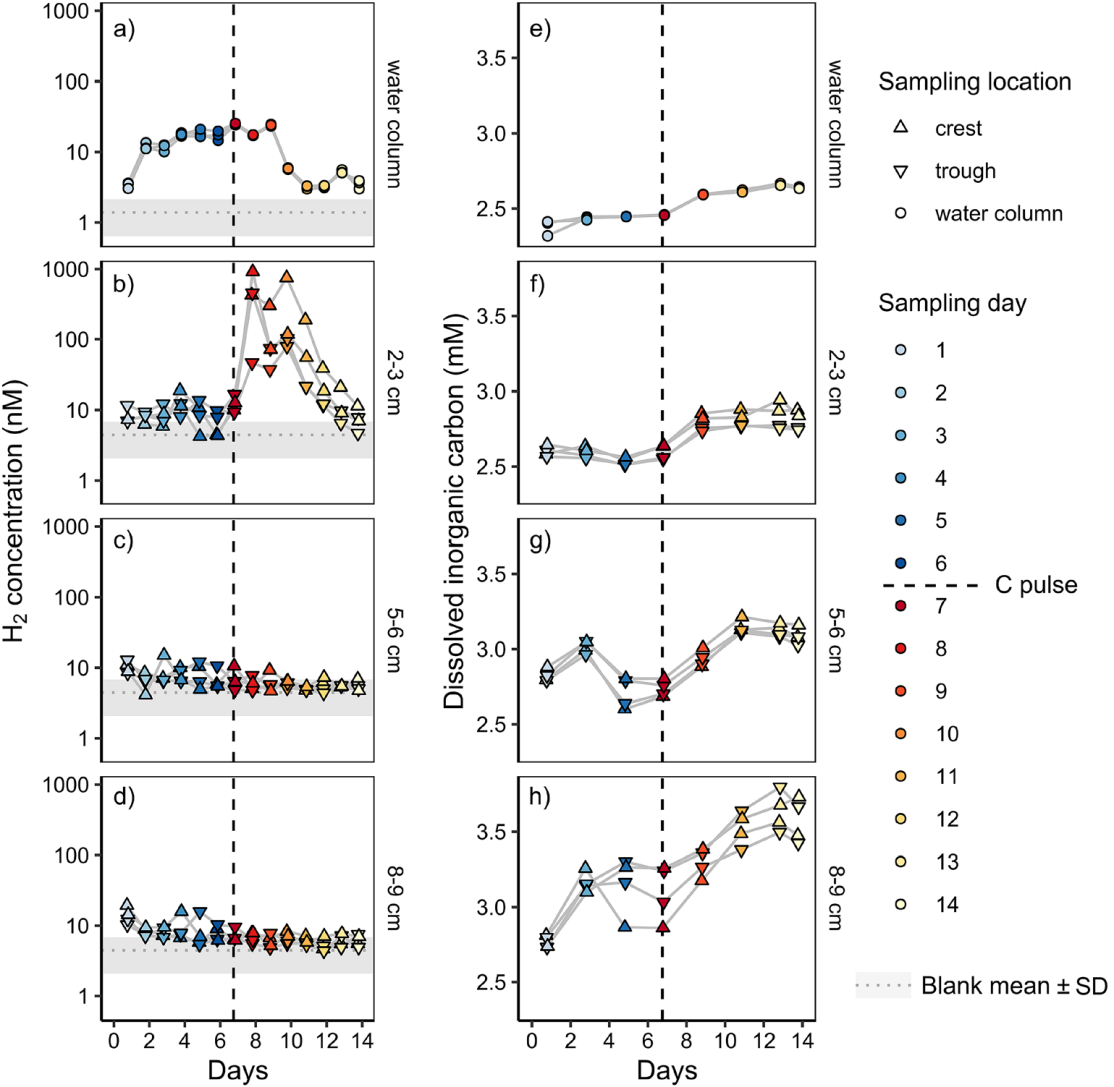
Hydrogen (**a**–**d**) and DIC concentrations (**e**–**h**) in the water column (**a**, **e**) and sediment (**b**–**d**, **f**–**h**) during a flume experiment with permeable sediment from Middle Park Beach. A pulse of organic material in the form of spirulina powder to approximate an equivalent of ~ 1 mM glucose was added to the water column on Day 7 of the experiment.). Note the logarithmic scale of the *y*‐axes in (**a**–**d**).

To bridge the gap between laboratory and field observations, we investigated H_2_ turnover in response to a pulse of organic material in a flume experiment under near‐natural flow conditions. Although the water column was oxygenated throughout the experiment, the sediment column rapidly turned anoxic and formed typical oxygen distribution patterns across the sculpted sand ripples (Supporting Information Fig. S4). During the initial stabilization phase of 7 d, H_2_ concentrations in all three targeted sediment depth horizons and the water column fluctuated around a baseline of around 10 nM (Fig. 6a–d). The addition of dried spirulina to approximate an equivalent concentration of 1 mM glucose on Day 7 led to an increase in H_2_ concentrations up to two orders of magnitude in the top 2–3 cm of sediment on Day 8 (Fig. 6b). High H_2_ concentrations prevailed for 3 more days until gradually receding back to baseline. Deeper sediment layers showed no increase in H_2_ concentrations after the carbon pulse, but rather a slight decrease of the baseline toward detection limit (Fig. 6c, d). It thus appears that fermenters rapidly responded to increased carbon availability, but only in the top few cm of sediment where the spirulina was likely to penetrate. This led to the stimulation of respiratory processes, presumably sulfate reduction, that consumed any excess H_2_ also in deeper layers. The production of DIC was clearly stimulated by carbon addition, including the deeper sediment layers (Fig. 6e–h).

## Conclusions

In conclusion, transient accumulation of H_2_ in permeable sediments and overlying waters is likely—associated to pulsed enrichment of labile organic matter. This can be induced by external inputs from the water column from natural processes such as coral spawning or settling of algal detritus. We were also able to induce H_2_ production experimentally within the sediment through vigorous mechanical disturbance and days of anoxia. We postulate both these processes led to organic matter release either via cell destruction (most likely microalgae) in the case of disturbance, or death of facultative anaerobes and or viral lysis after days of anoxia. Both these mechanisms have relevance within an environmental context as they provide insight into processes likely to occur both during storm events (mechanical disturbance) as well as prolonged calm (prolonged anoxia). Future studies of H_2_ dynamics in permeable sediments should therefore focus on events associated with organic matter inputs, disturbance and unusual calm conditions to better understand the in situ dynamics of H_2_.

## Conflict of Interest

None declared.

## Supporting information


**Fig. S1.** H_2_ and O_2_ concentrations from manual sediment cores processed in situ.
**Fig. S2.** Keeling plots of H_2_ concentration measured in overlying water at Heron Island in 2018 and 2019.
**Fig. S3.** DOC concentrations from slurry Experiments C1 and C2.
**Table S1.** Overview of slurry experiments and experimental treatments.

**Figure S4.** Supplementary video.

## Data Availability

All data are available upon request from the authors.
