# Supplementary material for: Pulses of labile carbon cause transient decoupling of fermentation and respiration in permeable sediments
Source: Limnol Oceanogr. 2023 Jul 31;68(9):2141–52. doi: 10.1002/lno.12411 (PMC10952694; doi:10.1002/lno.12411)
Supplement: Supplementary file 1 — Fig. S1. H2 and O2 concentrations from manual sediment cores processed in situ. Fig. S2. Keeling plots of H2 concentration measured in overlying water at Heron Island in 2018 and 2019. Fig. S3. DOC concentrations from slurry Experiments C1 and C2. Table S1. Overview of slurry experiments and experimental treatments. [file LNO-68-2141-s002.docx]

**Supplementary Material**

**Table S1: Overview of slurry experiments and experimental treatments. Each line represents one treatment consisting of triplicate serum vials. Sites are Middle Park Beach (MPB), Heron Island (HI), Hjerting Badehotel (HB) and Fællestrand (FS).**

|  | **Site** | **Sample depth** | **Seawater** | **Oxygen** | | **Disturbance ^a^** | **C addition** | **Inhibition** |
| --- | --- | --- | --- | --- | --- | --- | --- | --- |
|  |  |  |  | **Processing** | **Incubation** |  |  |  |
| **Exp. A** | MPB | 0-5 cm | Natural | Oxic | Anoxic | Shaken | - | - |
|  |  | 0-5 cm |  | Oxic | Anoxic-Oxic-Anoxic |  | Glucose on day 8 | - |
|  |  | 0-5 cm |  | Oxic | Anoxic |  | - | Autoclaved |
|  |  | 25-30 cm |  | Oxic | Anoxic |  | - | - |
|  |  | 25-30 cm |  | Anoxic | Anoxic |  | - | - |
|  |  | 25-30 cm |  | Anoxic | Anoxic |  | Glucose on day 8 | - |
|  |  | 25-30 cm |  | Anoxic | Anoxic-Oxic-Anoxic |  | - | - |
|  |  | 25-30 cm |  | Anoxic | Anoxic-Oxic-Anoxic |  | Glucose on day 8 | - |
| **Exp. B** | MPB | 0-30 cm | Natural | Oxic | Anoxic | Shaken | - | - |
|  | MPB |  |  |  |  | Shaken |  | Molybdate |
|  | MPB |  |  |  |  | Shaken |  | Autoclaved |
|  | MPB |  |  |  |  | Still |  | - |
|  | MPB |  |  |  |  | Still |  | Molybdate |
|  | MPB |  |  |  |  | Still |  | Autoclaved |
|  | HI |  |  |  |  | Shaken |  | - |
|  | HI |  |  |  |  | Still |  | - |
|  | HB |  |  |  |  | Shaken |  | - |
|  | HB |  |  |  |  | Still |  | - |
|  | FS |  |  |  |  | Shaken |  | - |
|  | FS |  |  |  |  | Still |  | - |
| **Exp. C1** | MPB | 0-30 cm | Artificial | Oxic | Anoxic | Still | - | - |
|  |  |  | Artificial |  |  | Shaken | - | - |
|  |  |  | Artf. w/o sulfate |  |  | Still | - | - |
|  |  |  | Artf. w/o sulfate |  |  | Shaken | - | - |
|  |  |  | Artf. w/o sulfate |  |  | Still | Glucose | - |
|  |  |  | Artf. w/o sulfate |  |  | Still | Spirulina | - |
|  |  |  | Artificial |  |  | Still | Glucose | - |
|  |  |  | Natural |  |  | Shaken | - | - |
|  |  |  | Natural |  |  | Shaken | - | Autoclaved |
| **Exp. C2** | MPB | 0-30 cm | Artf. w/o sulfate | Oxic | Anoxic | Still | - | - |
|  |  |  |  |  |  | Shaken 50 rpm |  |  |
|  |  |  |  |  |  | Shaken 200 rpm |  |  |
|  |  |  |  |  |  | Roller shaker |  |  |
| ^a^ Shaken at 130 rpm unless noted otherwise. | | | | | | | | |


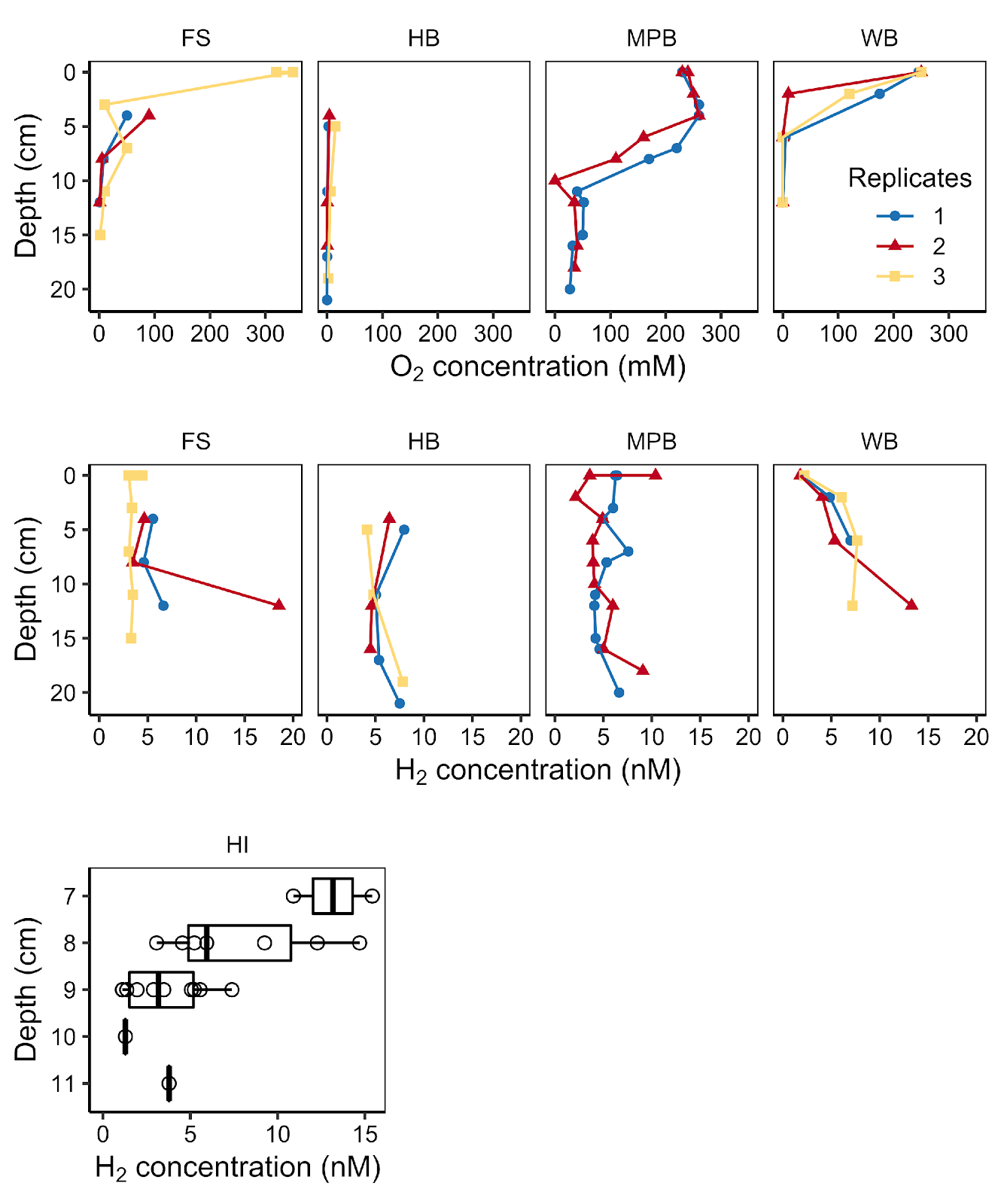


Fig. S1: H_2_ and O_2_ concentrations from manual sediment cores processed in situ. Sites are Fællestrand (FS), Hjerting Badehotel (HB), Middle Park Beach (MPB), Werribee Southern Beach (WB) and Heron Island (HI)


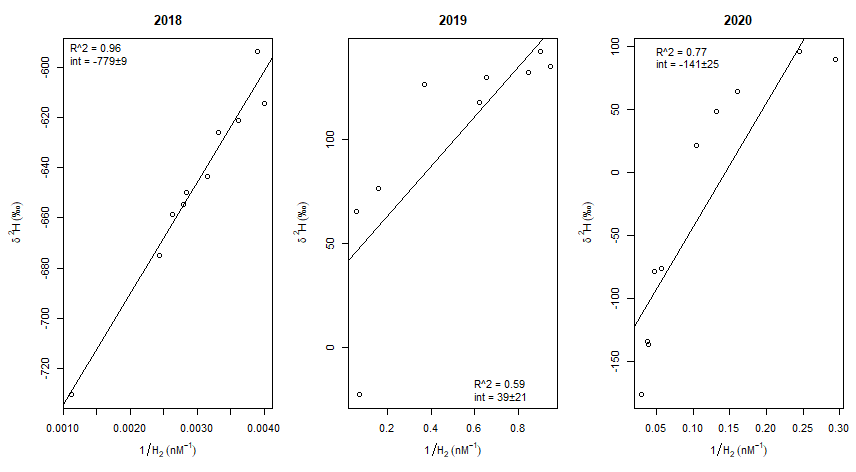


Fig. S2: Keeling plots of H_2_ concentration measured in overlying water at Heron Island in 2018 and 2019. p values for intercepts are 10^-12^, 0.1 and 0.0005 respectively. 2019 intercept is 73 +/- 10 (p = 0.003) if outlier is removed.


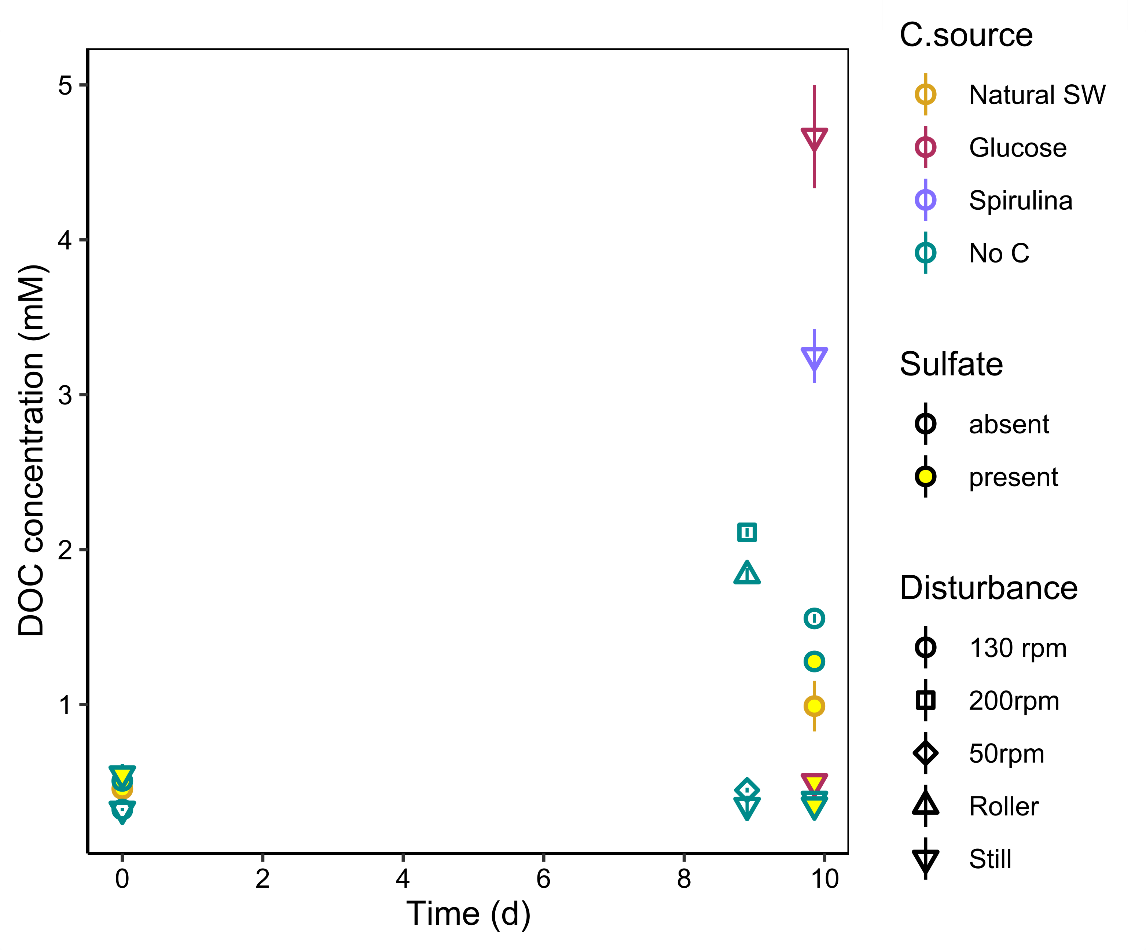


Fig. S3: Dissolved organic carbon (DOC) concentrations from slurry experiments C1 and C2. Samples were collected at the start and end of the experiments. Error bars represent standard error of the mean (n=3)

Figure S4 video file uploaded separately in supp material
